# Steroid Hydroxylation by Mutant Cytochrome P450 BM3-LG23 Using Two Expression Chassis

**DOI:** 10.3390/ijms262110728

**Published:** 2025-11-04

**Authors:** Veronika Poshekhontseva, Vera Nikolaeva, Andrey Shutov, Alexey Kazantsev, Olesya Sazonova, Nicolai Strizhov, Marina Donova

**Affiliations:** 1 G.K. Skryabin Institute of Biochemistry and Physiology of Microorganisms (IBPM), Federal Research Center “Pushchino Scientific Center for Biological Research of the Russian Academy of Sciences”, Russian Academy of Sciences, 142290 Pushchino, Russia; nikolae_vera@rambler.ru (V.N.); w___w@rambler.ru (A.S.); sazonova_oi@rambler.ru (O.S.); nicolai@strizhov.de (N.S.); mv_donova@rambler.ru (M.D.); 2 Chemistry Department, Lomonosov Moscow State University, 119991 Moscow, Russia; mak@org.chem.msu.ru

**Keywords:** cytochrome P450 BM3, *Escherichia coli*, *Mycolicibacterium smegmatis*, steroid, hydroxylation, bioconversion

## Abstract

The unique cytochrome P450 BM3 from *Priestia megaterium* (syn. *Bacillus megaterium*) is renowned for its versatile high catalytic activity. The *cyp102A1-LG23* gene encoding its CYP102A1-LG23 mutant variant was expressed in *Escherichia coli* and *Mycolicibacterium smegmatis*. The in vivo activity of the heterologous enzyme was assessed with respect to androstenedione (AD), androstadienedione (ADD), testosterone (TS) and dehydroepiandrosterone (DHEA). Alongside 7β-hydroxylation, the heterologous enzyme catalyzed the mono- and dihydroxylation of C19 steroids. For the first time, the formation of 7β-, 6β- and 11α-hydroxylated derivatives of ADD using a bacterial enzyme, as well as the hydroxylation of DHEA at the C7α and C7β positions, and its dihydroxylation with the formation of the 7α,15α-dihydroxylated derivative using the mutant cytochrome P450 BM3 were demonstrated. The steroid structures were confirmed using mass spectrometry and ^1^H NMR spectroscopy. The advantages of using mycolicibacteria as a bacterial chassis for gene expression were also shown. The results demonstrate the unusual properties of the mutant cytochrome P450 BM3-LG23 and open up prospects for its application in the biotechnological production of valuable hydroxysteroids.

## 1. Introduction

Stereo- and regiospecific hydroxylation of C-H bonds is one of among the most desirable reactions in steroid functionalization, ensuring the efficient production of therapeutically valuable hydroxysteroids [1]. The presence of oxygen-containing groups (most often hydroxyl) in the steroid molecule is responsible for the biological effects of steroids. The number, stereo- and regio positions of the introduced hydroxyl groups play an essential role. For instance, the presence of 11β-hydroxyl is crucial for the anti-inflammatory activity of steroids (hydrocortisone, prednisolone) [2], active forms of vitamin D3 contain 1α- and 25α-hydroxyl groups [3], cardioactive steroids have a 14β-hydroxyl group [4], and 27-hydroxycholesterol has demonstrated activity against SARS-CoV-2 and one of the causative agents, as well as HCoV-OC43, a common cold virus from the coronavirus family [5].

Steroids with a hydroxyl group at C7 attract special attention due to their neuroprotective, anti-inflammatory and immunological properties. For example, 3β,7α-dihydroxyandrost-5-ene-17-one (7α-OH-DHEA) is a well-known biologically active steroid with immunomodulatory properties that is widely used in medicine to treat rheumatoid arthritis and other autoimmune diseases [6,7]. C7β steroidal alcohols can be used to protect against acute and chronic neuronal damage induced by stroke, brain trauma and cerebral ischemia such as damage that may be caused by sub-arachnoid hemorrhage or that occurs during heart bypass surgery [8].

In addition, some C7β-alcohols can serve as precursors for the synthesis of other 7β-hydroxysteroids. Notably, the 7β-hydroxylated derivative of androst-4-ene-3,17-dione (AD) is used for the production of ursodeoxycholic acid (UDCA) [9,10], a key medication for treating various diseases of the hepatobiliary system [11,12,13], and also exhibits an adjuvant therapeutic effect in neurological disorders [14,15] and certain cancers [16,17]. Regio- and stereospecific hydroxylation of steroids by chemical means is rather complicated or even impossible and is rarely used in industry [1,18,19]. Microbial production of hydroxysteroids is a sustainable, cost-effective and eco-friendly alternative to chemical synthesis.

Cytochrome P450 monooxygenases (P450s or CYPs) play a key role in the oxyfunctionalization of steroids and are of particular interest for genetic and protein engineering. Cytochrome P450 BM3 (CYP102A1) is a well-known C12–C20 fatty acid hydroxylase from *Bacillus megaterium* (syn. *Priestia megaterium*) and one of the most active cytochrome P450 monooxygenases. This is due to its “self-sufficient” electron transfer pattern, mediated by the fusion of a P450 domain and a eukaryotic-like cytochrome P450 reductase (CPR) [20,21,22].

To date, P450 BM3 mutants with an expanded substrate specificity and various activity patterns have been obtained through rational design or directed evolution [23,24]. For example, the mutant P450 BM3 (F87A) has been developed to perform hydroxylation of testosterone at the 2β- and 15β-position [25]. Li et al. constructed an LG23 mutant with 14 mutations in the heme part of the P450 BM3 enzyme that hydroxylated androstane steroids at the C7β position [26].

Previously, we obtained promising recombinant *Mycolicibacterium smegmatis* strains that heterologously expressed *cyp102A1-LG23* [27]. These strains exhibited significant 7β-hydroxylating activity in vivo, producing 7β-OH-AD as the major AD bioconversion product. 1β-Hydroxy androstenedione (1β-OH-AD) was also identified among the by-products. It is noteworthy that hydroxylation of steroids at the 1β-position is an extremely rare reaction and that the production of potentially valuable new bioactive 1β-hydroxysteroids may be of interest to the pharmaceutical and biomedical industries.

This work aimed to investigate the in vivo bioconversion of various androstane steroids using recombinant *E. coli* and *M. smegmatis* strains heterologously expressing *cyp102A1-LG23* and identify compounds with the potential to be developed into new drugs. The results could inform the development of new drug candidates and the microbial production of valuable hydroxylated steroid synthons using cytochrome P450 BM3.

## 2. Results

**Figure 1:**
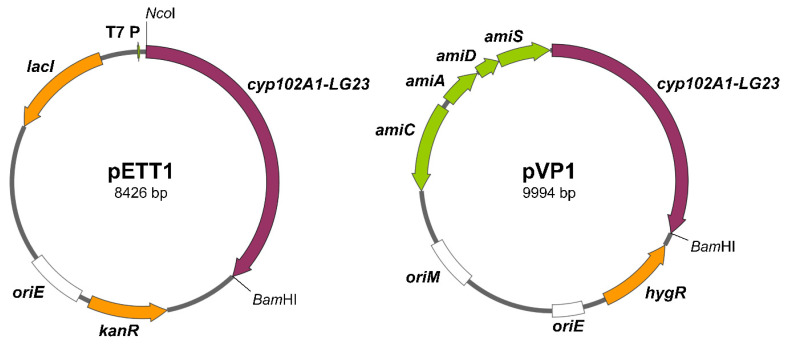
Maps of the pETT1 and pVP1 plasmids, containing the synthetic *cyp102A1-LG23* gene. T7 P—the T7 RNA-polymerase promoter; *amiC*, *amiA, amiD*, *amiS*—components of the acetamidase promoter; *kanR*—kanamycin resistance marker; *hygR*—hygromycin resistance marker; *oriE*—origin of replication in *E. coli*; *oriM*—origin of replication in *Mycolicibacterium*; *lacI*—lac repressor gene. Recognition sites the *Nco*I and *Bam*HI restriction endonucleases used for cloning are indicated.

The pETT1 plasmid was obtained in this work and the pVP1 plasmid was constructed in our previous study [27] (Figure 1). The recombinant *Escherichia coli* BL21 (DE3) (pETT1) and *Mycolicibacterium smegmatis* BD (pVP1) strains were used to evaluate the functionality of the cytochrome P450 BM3-LG23 mutant in its ability to hydroxylate C19 steroids.

The hydroxylating activity of the transformants expressing the *cyp102a1-LG23* gene was tested against four androstane steroids: AD, ADD, TS and DHEA. The strains transformed with the empty plasmids pET28a or pMyNTA were used as a control.

### 2.1. Expression of cyp102A1-LG23

**Figure 2:**
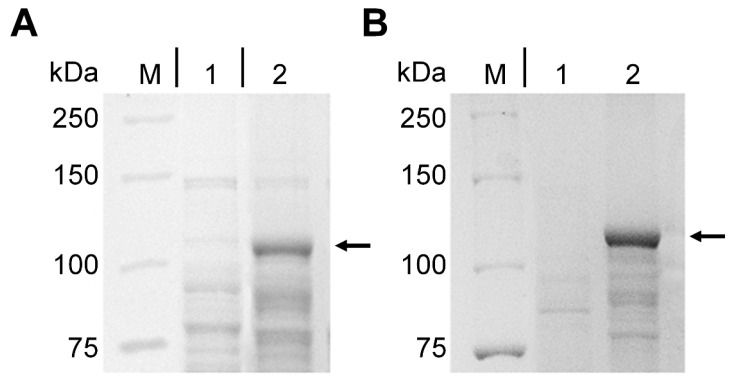
SDS-PAGE analysis of P450 BM3-LG23. Lane M: protein marker (Bio-Rad, Hercules, CA, USA); Lane 1: lysates of induced *E. coli* BL21 (DE3) (pET28a) (empty vector); Lane 2: lysates of induced *E. coli* BL21 (DE3) (pETT1) (**A**). Lane M: protein marker; Lane 1: lysates of induced *M. smegmatis* BD (pMyNTA) (empty vector); Lane 2: lysates of induced *M. smegmatis* BD (pVP1) (**B**). The arrows indicate the positions of P450 BM3-LG23.

Following induction of expression, the presence of cytochrome P450 BM3-LG23 was analyzed using SDS-PAGE. As shown in Figure 2, the target enzyme produced a clear band in both recombinants. The molecular weight of the protein was approximately 118 kDa in both cases, which corresponds to the theoretically expected value.

### 2.2. Bioconversion of AD

During the in vivo conversion of androstenedione (AD) by the *E. coli* BL21 (DE3) (pETT1) and *M. smegmatis* BD (pVP1) strains, the formation of the major product (retention time (RT) of 12.34 min, 303 *m*/*z*) and the minor product (RT 18.78 with 303.1 *m*/*z*) was observed. These compounds were identified as 7β-OH-AD and 1β-OH-AD, respectively by ^1^H NMR spectroscopy.

In addition, the compound (RT 4.96 min, 318.9 *m*/*z*) was indicated in small amounts in the *M. smegmatis* culture broth and identified as 1β,7β-dihydroxyandrostenedione (1β,7β-diOH-AD). This compound was not detected in the case of *E. coli*. The yield of hydroxylated products in *M. smegmatis* was several times higher than in *E. coli* (Table 1 and Table 2).

### 2.3. Bioconversion of ADD

The hydroxylation of androstadienedione (ADD) occurred with less regioselectivity in both strains. The major bioconversion product was 7β-OH-ADD (RT 10.71, 300.9 *m*/*z*), which was present in significantly higher amounts in the *M. smegmatis* BD (pVP1) strain.

Along with 7β-OH-ADD, other monohydroxy derivatives were formed such as 6β-OH-ADD (RT 12.83, 300.8 *m*/*z*) and 11α-OH-ADD (RT 9.29, 300.9 *m*/*z*). Additionally, trace and small amounts of 7β-OH-AD were identified in the culture broth of *E. coli* BL21 (DE3) (pETT1) and *M. smegmatis* BD (pVP1), respectively. The formation of the dihydroxylated product 6β,11α-diOH-ADD (316.8 *m*/*z*, RT 4.79) was only noted in the case of ADD bioconversion with recombinant *M. smegmatis* (Table 1 and Table 2).

### 2.4. Bioconversion of Testosterone

Both recombinant strains hydroxylated testosterone (TS) at positions C7β and C15β, forming 7β-OH-TS (RT 9.49, 305.1 *m*/*z*) and 15β-OH-TS (RT 10.64, 305 *m*/*z*), respectively. However, the *M. smegmatis* strain produced higher levels of 7β-OH-AD and 1β-OH-AD (5 and 2 times higher, respectively) than hydroxylated testosterone derivatives (Table 1 and Table 2).

### 2.5. Bioconversion of DHEA

Compared to 3-keto-Δ4-steroids (AD, ADD and TS), a feature of the bioconversion of DHEA was the formation of its stereoisomer, 7α-OH-DHEA (RT 3.58), along with the 7β-hydroxy derivative (7β-OH-DHEA, RT 3.36) in both strains.

Also of interest is the accumulation of a dihydroxy derivative with axial hydroxyl groups—7α,15α-diOH-DHEA (RT 2.99). Trace amounts of 7β-OH-AD were only detected in the *M. smegmatis* BD (pVP1) culture broth (Table 1 and Table 2).

In general, the strain obtained on the *M. smegmatis* chassis was significantly more efficient than the *E. coli* recombinant in terms of steroid bioconversion (Table 1).

## 3. Discussion

Microbial cytochrome P450 monooxygenases are widely used in various fields of biotechnology and synthetic biology [23]. CYP102A1 was the third cytochrome P450 to be isolated and characterized from *Bacillus megaterium* (syn. *Priestia megaterium*) [28], and is therefore often referred to as P450 BM3 in the literature. This enzyme is currently recognized as the most active among the known cytochromes P450, performing oxyfunctionalization of inactive C-H bonds. Using protein engineering and directed evolution methods, various enzyme variants have been obtained that exhibit activity against a wide range of organic molecules, including steroids such as estradiol [29], testosterone and progesterone [25,30], norandrostenedione [31], etc. In particular, mutant variants of CYP102A1 have been obtained that effectively catalyze the hydroxylation of testosterone and progesterone at the C2β and C15β positions [25]. The P450 BM3-LG23 mutant variant, which has 14 mutations in the heme part of the enzyme, hydroxylated testosterone at the C7β position [26]. This reaction was first detected using the triple mutant F87G/A328G/A330W (about 3% selectivity towards testosterone). This mutant served as the “starting template” in the construction of the polymutant variant P450 BM3-LG23 [26]. The catalytic efficiency of its synthetic analogue was demonstrated in the 7β-hydroxylation of AD by the *M. smegmatis* recombinant in vivo [27].

In this study, we focused on the unusual properties of CYP102A1-LG23. We demonstrated the functional activity of the heterologous P450 BM3-LG23 in vivo in the bioconversion of four steroid substrates (AD, ADD, TS, and DHEA) in recombinant *E. coli* and *M. smegmatis* strains.

**Figure 3:**
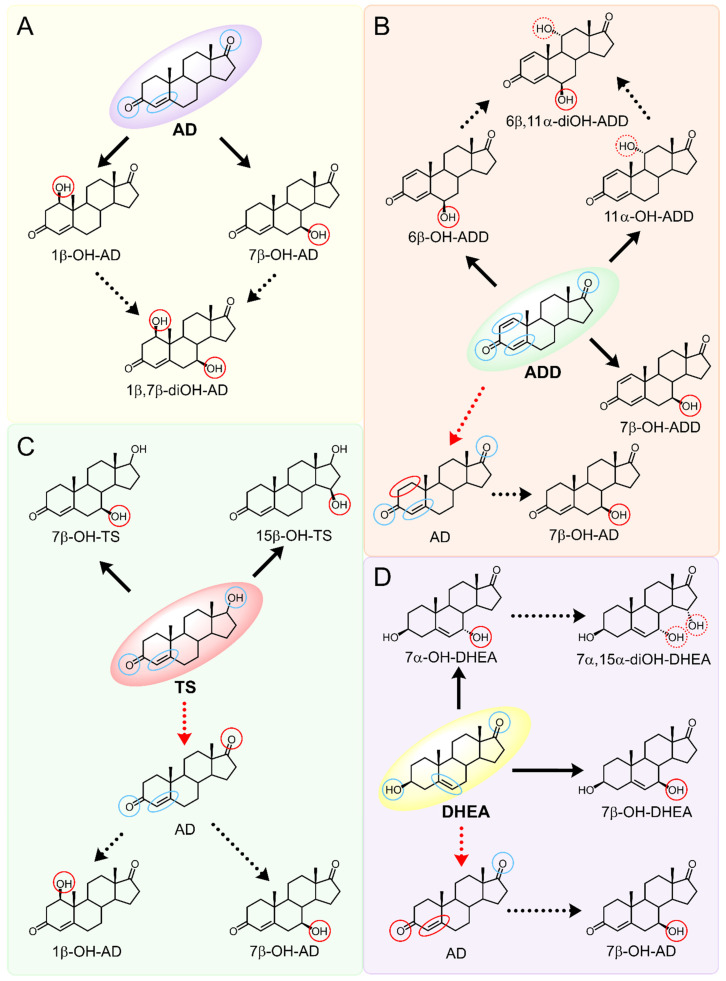
The hydroxylation of steroids by the P450 BM3-LG23 mutant in vivo in *M. smegmatis* BD (pVP1): androstenedione (AD) (**A**), androstadienedione (ADD) (**B**), testosterone (TS) (**C**), dehydroepiandrosterone (DHEA) (**D**). Black continuous arrows indicate reactions carried out by P450 BM3-LG23; black dotted arrows indicate putative bioconversion pathways; red dotted arrows indicate reactions carried out by the strain’s own enzymes—17β-HSD and 1(2)-reductase; blue ovals indicate the location of double bonds in the substrate molecule; red ovals indicate changes to the location of double bonds in the product molecule; blue circles indicate the location of hydroxyl/keto groups in the substrate molecule; red continuous circles indicate the sites of β-hydroxylation in the substrate molecule and the β-OH group position in the product molecule; red dotted circles indicate the sites of α-hydroxylation in the substrate molecule and the α-OH group position in the product molecule.

As we noted earlier [27] and confirmed in the present study, alongside the formation of 7β-OH-AD formed as the major product from AD, the accumulation of a compound that we identified as 1β-OH-AD was observed, as well as a 1β,7β-dihydroxylated derivative of AD (Figure 3). This derivative had not previously been described for similar reactions.

It should be noted that the enzymatic introduction of a hydroxyl group into the apical 1β-position is a rather rare reaction. The formation of 1β-OH-AD has only previously been described for fungal cultures [32]. Selective 1β-hydroxylation of lithocholic acid has been described for one of the CYP102A1 mutant variants [33], and 1β-hydroxytestosterone has been identified as one of the monohydroxylated metabolites formed by P450 BM3 mutants from testosterone [30]. It is noteworthy that other P450 BM3 mutants, such as M01A82W and M11A82W 82W [34], as well as mutant 139-3, carried out hydroxylation of AD at the stereoisomeric 1α position [35]. According to the authors’ study [35], the introduction of a hydroxyl group at the 1α position depends on the presence of the native amino acid residue Arg379 in the enzyme’s active centre, and mutation of this residue (R379S) results in loss of this function. The obtained data indicate that including targeted mutations into the heme part of cytochrome P450 BM3 changes the stereoposition of the introduced hydroxyl groups.

Regio- and stereoselectivity of hydroxylation catalyzed by CYP102A1-LG23 depended significantly on the structure of the steroid substrate (Figure 3) and on the microbial recipient used for expression (Table 1).

Thus, during the hydroxylation of DHEA, which differs from AD by the presence of a 3β-hydroxyl group, along with a 7β-hydroxylated derivative, its 7α-hydroxy isomer was formed. The major product from DHEA was the 7α,15α-dihydroxylated derivative (7α,15α-diOH-DHEA) (Table 1, Figure 3), which is a valuable intermediate in the synthesis of the popular drug—drospirenone. Previously, the ability to form this compound had only been described for fungal hydroxylases [36,37], and the mono- and dihydroxylation of DHEA by the mutant variant of P450 BM3 was demonstrated for the first time in this work (Table 1, Figure 3).

The obtained data are inconsistent with those published for other P450 BM3 mutants, i.e., M01A82W, M11A82W, and M01A82WS721 [34]. These three mutants hydroxylated 3-keto-4-ene steroids, such as testosterone and methyltestosterone but exhibited no activity against 3β-hydroxy-5-ene steroids, including DHEA.

The presence of an additional double bond (C1–C2) in the ADD molecule significantly affected the selectivity of hydroxylation. In contrast to the hydroxylation of AD, the metabolites identified included the 7β-hydroxy derivative, as well as C6β- and C11α-alcohols (Table 1, Figure 3). The formation of 7β-OH-ADD, 6β-OH-ADD, and 11α-OH-ADD using bacterial enzymes has not previously been described in the literature.

Unlike ADD and DHEA, testosterone is frequently used as a substrate in studies assessing the steroid-transforming activity of P450 BM3 mutant variants. As in other studies [26,30], the 7β-monohydroxylated derivative of testosterone was identified as a metabolite of the mutant P450 BM3 LG23 (Table 1, Figure 3).

This study also provides the first comparative evaluation of using two different bacterial chassis for expressing *cyp102A1-LG23*: *E. coli and M. smegmatis*. The use of mycolicibacteria, which possess efficient steroid transport systems and carry out oxidative degradation of sterol side chains, opens up prospects for the one-step production of valuable hydroxylated steroids from readily available and inexpensive phytosterols. Zhao et al. [38] first demonstrated the feasibility of this approach, showing that expressing a mutant *cyp102A1* in *M. neoaurum* allowed the production of 7β-OH-AD from phytosterol.

Our results showed that expression of *cyp102A1-LG23* in mycolicibacteria resulted in the more efficient production of hydroxy derivatives than in *E. coli* (Table 1). For example, the production of 7α,15α-diOH-DHEA in *M. smegmatis* was 15 times higher than that in *E. coli*. Overall, the efficiency of steroid transformation in recombinant mycolicibacteria was 1.6–15 times higher (Table 1). The higher degree of steroid conversion in vivo in mycolicibacteria appears to be due to more efficient steroid transport than in *E. coli*. This is also confirmed by the high yield of hydroxylated products obtained through the in vitro transformation of steroids by P450 BM3-LG23 in *E. coli* lysates.

Mycolicibacteria are known to have an ABC-like steroid transport system, which is encoded by the mce4 operon genes. It is involved in the transport of cholesterol and closely related compounds [39,40]. The cell wall of *Mycolicibacterium* contains mycolic acids (up to 60% of its weight), making it highly hydrophobic [41], which allows these bacteria to effectively oxidize a wide range of hydrophobic compounds [42,43].

The spectrum of metabolites produced by P450 BM3-LG23 in mycolicibacteria also differed somewhat from that in *E. coli* (Table 1). Thus, during the bioconversion of ADD by *M. smegmatis* BD (pVP1) 7β-OH-AD was detected among the metabolites. This compound is apparently formed by the reduction of the C1–C2 double bond in ADD, forming AD, which is then 7β-hydroxylated using P450 BM3-LG23 (the ADD → AD → 7β-OH-AD route), but not via the 7β-OH-ADD → 7β-OH-AD route, as traces of AD were found in the culture broth (Figure 3). The presence of 1-ene reductase activity in mycolicibacteria has been described previously [44].

In turn, during the bioconversion of TS by recombinant mycolicibacteria, the formation of 1β- and 7β-OH-AD was observed, which was not observed in the recombinant *E. coli* (Table 1). This may be due to the oxidation of the 17β-hydroxy group of testosterone to AD, which is then hydroxylated by the mutant P450 BM3-LG23 (TC → AD → 7β/1β-OH-AD) (Figure 3). This is supported by the detection of trace amounts of AD in the medium during TS bioconversion, and the absence of 1β-OH-TS among the metabolites. The presence of 17β-hydroxysteroid dehydrogenase activity in mycolicibacteria and its reversibility are well documented [44,45].

Presumably, P450 BM3-LG23 may also be involved in the oxidation of TC to AD via a germinal diol intermediate, as described for 3-keto-5β-cholanic acid [46], and as supported by the presence of trace amounts of 1β- and 7β-OH-AD in an in vitro *E. coli* cell homogenate reaction. A similar mechanism may be employed in the synthesis of 7β-OH-AD from DHEA.

## 4. Materials and Methods

### 4.1. Chemicals

Androst-4-ene-3,17-dione (androstenedione, AD), androsta-1,4-diene-3,17-dione (androstadienedione, ADD), 17β-hydroxyandrost-4-ene-3-one (testosterone, TS), 3β-hydroxyandrost-5-ene-17-one (dehydroepiandrosterone, DHEA) and acetamide (AcA) were purchased from Sigma-Aldrich (St. Louis, MO, USA). 3β,7α-Dihydroxyandrost-5-ene-17-one (7α-OH-DHEA) was obtained from Schering AG (Berling, Germany); 3β,7β-Dihydroxyandrost-5-ene-17-one (7β-OH-DHEA) (over 97% purity)—from the Skryabin Institute of Biochemistry and Physiology of Microorganisms RAS (IBPM RAS). Hygromycin, yeast extract and peptone were purchased from Panreac (Castellar del Vallès, Spain); isopropyl-β-D-1-thiogalactopyranoside (IPTG)—from AppliChem GmbH (Darmstadt, Germany), methyl-β-cyclodextrin (MCD)—from Wacker Chemie (Munich, Germany), Tween-80—from Serva (Heidelberg, Germany), silica gel—from Fluka (Everett, WA, USA). Other materials were of reagent grade and purchased from domestic companies.

### 4.2. Strains and Plasmids

*Escherichia coli* BL21 (DE3) and *Mycolicibacterium smegmatis* BD [47] were used to express the *cyp102A1-LG23* gene encoding the mutant P450 BM3-LG23. The nucleotide sequences of the wild-type *cyp102A1* gene were retrieved from KEGG (BG04_163) and GenBank (ACCESSION J04832, VERSION J04832.1). The amino acid sequence of the P450 BM3-LG23 heme fragment—from RCSB PDB (PDB ID: 6LY4). The target mutant gene was synthesized by GenScript (Piscataway, NJ, USA) and ligated into the expression vectors pET28a and pMyNTA [27] to produce the recombinant plasmids pETT1 and pVP1 [27], respectively. For expression in the *Micolicibacterium* cells, the gene was codon-optimized. The plasmids harboring the synthetic *cyp102A1-LG23* gene were transferred into the competent cells of *E. coli* and *M. smegmatis* by transformation and electroporation [48], respectively. The transformants were selected on LB agar containing 50 μg/mL kanamycin and M3 agar containing 75 μg/mL hygromycin, respectively.

### 4.3. Heterologous Expression

The recombinant *E. coli* BL21 (DE3) strain harboring the ETT1 plasmid was incubated in a Luria–Bertani (LB) liquid medium supplemented with 50 μg/mL kanamycin. The recombinant *M. smegmatis* BD strain with the VP1 plasmid was incubated in the M3 liquid medium [47] with 50 μg/mL hygromycin. After incubation at 37 °C and 200 rpm for 12–15 h, 1 mL of each culture was inoculated into 50 mL of suitable fresh medium. When the *E. coli* strain reached an OD_600_ of 0.6 to 0.8, expression was induced with 0.2 mM IPTG. When an OD_600_ of the *M. smegmatis* strain reached 0.8–1, acetamide was added to a final concentration of 2 g/L to induce expression. Cultivation was then continued at 25 °C for 24 or 48 h, respectively.

The cells were separated by centrifugation at 5000× *g* for 15 min, resuspended in a cold potassium phosphate buffer (50 mM KH_2_PO_4_, 50 mM NaCl, pH 7.4) and disrupted by sonication on ice. The crude enzyme was analyzed by SDS–PAGE.

### 4.4. Steroid Bioconversion

The seed cultures of the recombinant *E. coli* BL21 (DE3) (pETT1) and *M. smegmatis* BD (pVP1) strains were grown at 37 °C and 200 rpm for 12–15 h in LB containing 50 μg/mL kanamycin or the M3 medium containing 50 μg/mL hygromycin, respectively.

For steroids bioconversion by the *E. coli* BL21 (DE3) (pETT1) strain, the Terrific Broth (TB) medium [49] with kanamycin (50 μg/mL) was used. Steroid transformation by the recombinant *M. smegmatis* BD (pVP1) strain was performed in the M3 medium containing hygromycin (50 μg/mL). The transformation media were inoculated with 2% (*v/v*) seed culture of the corresponding strain. Expression induction was performed as described above. Steroids (AD, ADD, TS or DHEA) were added as a suspension with MCD to a final concentration of 1 g/L. The substrate/MCD molar ratio was 1:1.5–1:3, mol/mol. Bioconversion was conducted aerobically at 25 °C and 200 rpm for 72 h and monitored by HPLC as described below.

### 4.5. Thin Layer Chromatography (TLC)

The steroids were extracted with a double volume of ethyl acetate (EtOAc). The extracts were applied onto an ALUGRAM SIL G/UV254 chromatographic plate (Macherey-Nagel, Düren, Germany), developed in a mixture of benzene–acetone (2:1, *v*/*v*) and visualized under UV-light (254 nm) on a CN-15MC UV Darkroom (Vilber Lourmat, Collégien, France). Additionally, to visualize hydroxylated steroids in UV light at 365 nm the TLC plates were stained using a MnCl_2_ reagent (MnCl_2_ × 4H_2_O—0.2 g, H_2_O—30 mL, ethanol—30 mL, H_2_SO_4_ (97%)—2 mL (dropwise)) and heating to 110 °C for 10 min.

### 4.6. High Performance Liquid Chromatography (HPLC)

An aliquot of the culture liquid was diluted tenfold with a 50% aqueous acetonitrile solution and centrifuged at 12,100× *g* for 8 min. The resulting supernatant was analyzed using an Agilent Infinity 1260 chromatography system (Agilent Technologies, Santa Clara, CA, USA) with a Symmetry RP-18 column (5 µm, 4.6 × 250 mm) and a Symmetry RP-18 precolumn (5 µm, 3.9 × 20 mm) (Waters, Milford, MA, USA). The mobile phases composition: (I) solution A, acetonitrile—THF (tetrahydrofuran)—water (10:10:80, *v*/*v*); solution B, 100% acetonitrile; gradient elution (from 0 to 14 min: solution A—100; from 14 to 28 min: solution B—0–60%). (II) acetonitrile—water—acetic acid (52:48:0.01, *v*/*v*). The flow rate was 1 mL/min; the column thermostat temperature was 50 °C. Steroids were detected at 254 (I) and 200/240 (II) nm.

### 4.7. Steroid Isolation

A culture broth was centrifuged at 4 °C and 27,300× *g* for 1 h. After the supernatant was extracted with EtOAc (70 mL) three times. The pooled ethyl acetate extract was evaporated under reduced pressure until dry. The steroids were separated by column chromatography on silica gel (Silicagel 90, 0.2–0.5 mm) (Fluka, Everett, WA, USA), and the steroid bioconversion products were isolated via stepwise elution with hexane—EtOAc –ethanol, as previously described [27]. The resulting fractions were evaporated until dry, dissolved in 500 µL of EtOAc, and further purified via TCL.

### 4.8. Mass-Spectrometry (MS) and ^1^H NMR Spectroscopy

MS spectra were registered on a Thermo Finnigan LCQ Advantage MAX quadrupole mass spectrometer (Thermo Fisher Scientific, Waltham, MA, USA) in [M + H]^+^ positive ion mode at an evaporator and capillary temperatures of 350 and 170 °C, respectively. MS/MS spectra were obtained using Normalized Collision Energy^TM^ at a range of 20–40%.

^1^H-NMR spectra were recorded using a Bruker Avance 400 spectrometer (Bruker, Bremen, Germany) at 400 MHz. Chemical shifts were measured relative to tetramethylsilane. Only the characteristic signals of steroids in the ^1^H-NMR are given.

The ^1^H-NMR spectral data of steroid bioconversion metabolites:

7β-Hydroxyandrost-4-ene-3,17-dione (7β-hydroxyandrostenedione, 7β-OH-AD): ^1^H NMR (CDCl_3_) δ: 5.78 (s, 1H, H-4), 3.60 (dt, *J* = 10.3, 5.2 Hz, 1H, H-7α), 1.24 (s, 3H, H-19), 0.95 (s, 3H, H-18).

1β-Hydroxyandrost-4-ene-3,17-dione (1β-hydroxyandrostenedione, 1β-OH-AD): ^1^H NMR (CDCl_3_) δ: 5.82 (d, *J* = 1.1 Hz, 1H, H-4), 4.06 (dd, *J* = 8.2, 7.6 Hz, 1H, H-1α), 1.27 (s, 3H, H-19), 0.93 (s, 3H, H-18).

1β,7β-Dihydroxyandrost-4-ene-3,17-dione (1β,7β-dihydroxyandrostenedione, 1β,7β-diOH-AD): ^1^H NMR (CDCl_3_) δ: 5.83 (br. s, 1H, H-4), 4.03 (dd, *J* = 9.2, 6.2 Hz, 1H, H-1α), 3.63–3.55 (m, 1H, H-7α), 1.30 (s, 3H, H-19), 0.95 (s, 3H, H-18).

6β-Hydroxyandrosta-1,4-diene-3,17-dione (6β-hydroxyandrostadienedione, 6β-OH-ADD): ^1^H NMR (CDCl_3_) δ: 7.05 (d, *J* = 10.2 Hz, 1H, H-1), 6.23 (dd, *J* = 10.2, 1.9 Hz, 1H, H-2), 6.18 (d, *J* = 1.9 Hz, 1H, H-4), 4.60 (t, *J* = 2.9 Hz, 1H, H-6α), 1.47 (s, 3H, H-19), 0.98 (s, 3H, H-18).

7β-Hydroxyandrosta-1,4-diene-3,17-dione (7β-hydroxyandrostadienedione, 7β-OH-ADD): ^1^H NMR (CDCl_3_) δ: 7.06 (d, *J* = 10.2 Hz, 1H, H-1), 6.25 (dd, *J* = 10.2, 1.9 Hz, 1H, H-2), 6.12 (br. s, 1H, H-4), 3.58 (td, *J* = 10.4, 5.4 Hz, 1H, H-7α), 1.30 (s, 3H, H-19), 0.97 (s, 3H, H-18).

11α-Hydroxyandrosta-1,4-diene-3,17-dione (11α-hydroxyandrostadienedione, 11α-OH-ADD): ^1^H NMR (CDCl_3_) δ: 7.78 (d, *J* = 10.3 Hz, 1H, H-1), 6.16 (dd, *J* = 10.3, 1.9 Hz, 1H, H2), 6.10 (br. s, 1H, H-4), 4.12 (td, *J* = 10.5, 5.1 Hz, 1H, H-11β), 1.34 (s, 3H, H-19), 0.97 (s, 3H, H-18).

6β,11α-Dihydroxyandrosta-1,4-diene-3,17-dione (6β,11α-dihydroxy androstadienedione, 6β,11α-diOH-ADD): ^1^H NMR (CDCl_3_) δ: 7.86 (d, *J* = 10.3 Hz, 1H, H-1), 6.18 (d, *J* = 2.0 Hz, 1H, H-4), 6.15 (dd, *J* = 10.3, 2,0 Hz, 1H, H-2), 4.55 (t, *J* = 2.8 Hz, 1H, H-6α), 4.18–4.11 (m, 1H, H-11β), 1.55 (s, 3H, H-19), 0.99 (s, 3H, H-18).

7β,17β-Dihydroxyandrost-4-ene-3-one (7β-hydroxytestosterone, 7β-OH-TS): ^1^H NMR (CDCl_3_) δ: 5.77 (br. s, 1H, H-4), 3.64 (t, *J* = 8.7 Hz, 1H, H-17α), 3.50–3.42 (m, 1H, H-7α), 1.23 (s, 3H, H-19), 0.82 (s, 3H, H-18).

15β,17β-Dihydroxyandrost-4-ene-3-one (15β-hydroxytestosterone, 15β-OH-TS): ^1^H NMR (CDCl_3_) δ: 5.75 (br. s, 1H, H-4), 4.23–4.17 (m, 1H, H-15α), 3.56 (t, *J* = 8.7 Hz, 1H, H-17α), 1.23 (s, 3H, H-19), 1.06 (s, 3H, H-18).

The MS/MS spectra are presented in Supplementary Figure S2.

Spectral data of DHEA hydroxylation products were obtained and described by us earlier [50].

### 4.9. Statistical Data Processing

The experimental data were obtained in three biological replicates. The results are presented as the mean ± standard deviation.

## 5. Conclusions

Sterol-transforming mycolicibacteria provide a convenient platform for expressing genes encoding mutant variants of cytochrome P450 BM3, which allows for the more efficient production of novel hydroxylated steroids than is possible with *E. coli*. Furthermore, combining the activity of heterologous mutant cytochrome P450 BM3 with the native enzyme systems of mycolicibacteria opens up prospects for the one-step production of valuable hydroxylated steroids from natural sterols.

## Figures and Tables

**Table 1:** Steroid bioconversion by *E. coli* BL21 (DE3) (pETT1) and *M. smegmatis* BD (pVP1).

Steroid Substrate	Transformation Product (TP)	Concentration (% mol.)		TP Ratio Ms/Ec
		*E. coli* (Ec)	*M. smegmatis* (Ms)	
Androstenedione (AD)	1β-OH-AD	0.62	6.05	9.8
	7β-OH-AD	11.78	26.55	2.3
	1β,7β-diOH-AD	n.d.	1.15	–
Androstadienedione (ADD)	6β-OH-ADD	1.76	11.54	6.6
	7β-OH-ADD	2.39	19.79	8.3
	11α-OH-ADD	1.94	8.96	4.6
	6β,11α-diOH-ADD	n.d.	7.25	–
	7β-OH-AD	trace	3.30	–
Testosterone (TS)	7β-OH-TS	0.65	2.07	3.2
	15β-OH-TS	1.10	1.79	1.6
	1β-OH-AD	n.d.	3.52	–
	7β-OH-AD	n.d.	9.62	–
Dehydroepiandrosterone (DHEA)	7α-OH-DHEA	0.29	1.93	6.7
	7β-OH-DHEA	0.49	2.33	4.8
	7α,15α-diOH-DHEA	0.32	4.97	15.5
	7β-OH-AD	n.d.	trace	–

The concentrations of the hydroxylation products were determined via HPLC (after 72 h of cultivation). n.d., not defined products. The dynamics of the accumulation of hydroxylation products in the recombinant strain cultures are shown in Supplementary Figure S1.

**Table 2:** Steroid substrates and their bioconversion products formed in vivo by P450 BM3-LG23 in *E. coli* BL21 (DE3) (pETT1) and *M. smegmatis* BD (pVP1).

Compound	RT (min)	*m*/*z*	Structure
Substrates			
Androst-4-ene-3,17-dione (AD)	26.36 (I)	n.d.	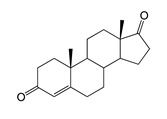
Androsta-1,4-diene-3,17-dione (ADD)	23.69 (I)	n.d.	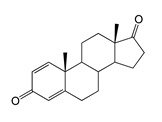
Testosterone (TS)	26.13 (I)	n.d.	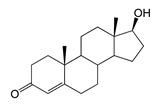
Dehydroepiandrosterone (DHEA)	8.49 (II)	n.d.	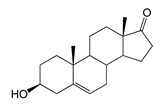
Bioconversion products			
1β-Hydroxyandrost-4-ene-3,17-dione (1β-OH-AD)	18.78 (I)	303	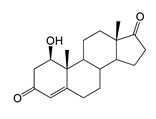
7β-Hydroxyandrost-4-ene-3,17-dione (7β-OH-AD)	12.34 (I)	303.1	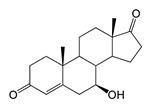
1β,7β-Dihydroxyandrost-4-ene-3,17-dione (1β,7β-diOH-AD)	4.96 (I)	318.9	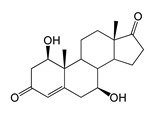
6β-Hydroxyandrosta-1,4-diene-3,17-dione (6β-OH-ADD)	12.83 (I)	300.8	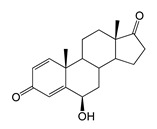
7β-Hydroxyandrosta-1,4-diene-3,17-dione (7β-OH-ADD)	10.71 (I)	300.9	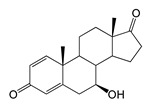
11α-Hydroxyandrosta-1,4-diene-3,17-dione (11α-OH-ADD)	9.28 (I)	300.9	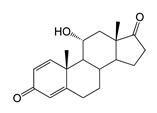
6β,11α-Dihydroxyandrosta-1,4-diene-3,17-dione (6β,11α-diOH-ADD)	4.79 (I)	316.8	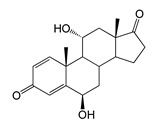
7β-Hydroxytestosterone (7β-OH-TS)	9.49 (I)	305.1	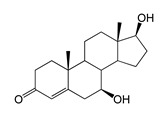
15β-Hydroxytestosterone (15β-OH-TS)	10.64 (I)	305	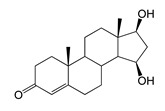
7α-Hydroxydehydroepiandrosterone (7α-OH-DHEA)	3.58 (II)	n.d.	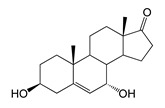
7β-Hydroxydehydroepiandrosterone (7β-OH-DHEA)	3.36 (II)	n.d.	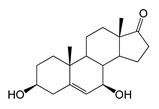
7α,15α-Dihydroxydehydroepiandrosterone (7α,15α-diOH-DHEA)	2.99 (II)	n.d.	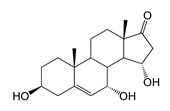

RT was determined via HPLC with method I or II, respectively. M/z was determined via mass spectrometry. n.d., not determined.

## Data Availability

Data is contained within the article and Supplementary Materials. Further inquiries can be directed to the corresponding author.

## Supplementary Materials

The following supporting information can be downloaded at https://www.mdpi.com/article/10.3390/ijms262110728/s1.

## References

[1] Pellissier H., Santelli M. (2001). Chemical and biochemical hydroxylations of steroids, a review. Org. Prep. Proced. Int..

[2] Berrie J.R., Williams R.A.D., Smith K.E. (1999). Microbial transformations of steroids-XI. Progesterone transformation by *Streptomyces roseochromogenes*—Purification and characterisation of the 16α-hydroxylase system. J. Steroid Biochem. Mol. Biol..

[3] Prosser D.E., Jones G. (2004). Enzymes involved in the activation and inactivation of vitamin D. Trends Biochem. Sci..

[4] Shah A.A., Ultanultan S.S., Adnan H.S. (2013). A whole-cell biocatalysis application of steroidal drugs. Orient. J. Chem..

[5] Marcello A., Civra A., Bonotto R.M., Alves L.N., Rajasekharan S., Giacobone C., Caccia C., Cavalli R., Adami M., Brambilla P. (2020). The cholesterol metabolite 27-hydroxycholesterol inhibits SARS-CoV-2 and is markedly decreased in COVID-19 patients. Redox Biol..

[6] Traish A.M., Kang H.P., Saad F., Guay A.T. (2011). Dehydroepiandrosterone (DHEA)—A precursor steroid or an active hormone in human physiology. J. Sex. Med..

[7] Kozłowska E., Urbaniak M., Kancelista A., Dymarska M., Kostrzewa-Susłow E., Łukasz S., Janeczko T. (2017). Biotransformation of dehydroepiandrosterone (DHEA) by environ-mental strains of filamentous fungi. RSC Adv..

[8] Wulfert E., Pringle A.K., Sundstrom L.E. (2002). Neuroprotective 7-Beta-Hydroxysteroids. Patent.

[9] Tonin F., Arends I.W.C.E. (2018). Latest development in the synthesis of ursodeoxycholic acid (UDCA): A critical review. Beilstein J. Org. Chem..

[10] Eggert T., Bakonyi D., Hummel W. (2014). Enzymatic routes for the synthesis of ursodeoxycholic acid. J. Biotechnol..

[11] Haal S., Guman M.S.S., Boerlage T.C.C., Acherman Y.I.Z., Brauw D.L.M., Bruin S., de Castro S.M.M., van Hooft J.E., van de Laar A.W.J.M., Moes D.E. (2021). Ursodeoxycholic acid for the prevention of symptomatic gallstone disease after bariatric surgery (UPGRADE): A multicentre, double-blind, randomised, placebo-controlled superiority trial. Lancet Gastroenterol. Hepatol..

[12] Byelyayeva N. (2015). Effectiveness of ursodeoxycholic acid (UDCA) in patients with chronic pancreatitis (CP) and biliary sludge. Pancreatology.

[13] Colombo C., Alicandro G., Oliver M., Lewindon P.J., Ramm G.A., Ooi C.Y., Alghisi F., Kashirskaya N., Kondratyeva E., Corti F. (2021). Ursodeoxycholic acid and liver disease associated with cystic fibrosis: A multicenter cohort study. J. Cyst. Fibros..

[14] Abdelkader N.F. (2020). Ursodeoxycholic and tauroursodeoxycholic acids as antiapoptotic agents: Modulation of Parkinson’s disease. Diagn. Manag. Park. Dis..

[15] Huang F. (2021). Ursodeoxycholic acid as a potential alternative therapeutic approach for neurodegenerative disorders: Effects on cell apoptosis, oxidative stress and inflammation in the brain. Brain Behav. Immun. Health.

[16] Goossens J.F., Bailly C. (2019). Ursodeoxycholic acid and cancer: From chemoprevention to chemotherapy. Pharmacol. Ther..

[17] Lugini A., Verna S., Buzzacchino F., Minelli M., Cristofani L. (2022). 1134P Prevention of hepatic toxicities associated with anaplastic lymphoma kinase inhibitors in the treatment of non-small cell lung cancer by administration of ursodeoxycholic acid: Analysis from the monoinstitutional analysis. Ann. Oncol..

[18] Loewenthal H. (1959). Selective reactions and modification of functional groups in steroid chemistry. Tetrahedron.

[19] Nassiri-Koopaei N., Faramarzi M.A. (2015). Recent developments in the fungal transformation of steroids. Biocatal. Biotransform..

[20] Munro A.W., Daff S., Coggins J.R., Lindsay J.G., Chapman S.K. (1996). Probing electron transfer in flavocytochrome P-450 BM3 and its component domains. Eur. J. Biochem..

[21] Munro A.W., Leys D.G., McLean K.J., Marshall K.R., Ost T.W.B., Daff S., Miles C.S., Chapman S.K., Lysek D.A., Moser C.C. (2002). P450 BM3: The very model of a modern flavocytochrome. Trends Biochem. Sci..

[22] Cirino P.C., Arnold F.H. (2003). A self-sufficient peroxide-driven hydroxylation biocatalyst. Angew. Chem. Int. Ed..

[23] Girvan H.M., Munro A.W. (2016). Applications of microbial cytochrome P450 enzymes in biotechnology and synthetic biology. Curr. Opin. Chem. Biol..

[24] Urlacher V.B., Girhard M. (2012). Cytochrome P450 monooxygenases: An update on perspectives for synthetic application. Trends Biotechnol..

[25] Kille S., Zilly F.E., Acevedo J.P., Reetz M.T. (2011). Regio- and stereoselectivity of P450-catalysed hydroxylation of steroids controlled by laboratory evolution. Nat. Chem..

[26] Li A., Acevedo-Rocha C.G., D’Amore L., Chen J., Peng Y., Garcia-Borràs M., Gao C., Zhu J., Rickerby H., Osuna S. (2020). Regio- and stereoselective steroid hydroxylation at C7 by cytochrome P450 monooxygenase mutants. Angew. Chem. Int. Ed..

[27] Poshekhontseva V.Y., Strizhov N.I., Karpov M.V., Nikolaeva V.M., Kazantsev A.V., Sazonova O.I., Shutov A.A., Donova M.V. (2023). Expression of Synthetic cyp102A1-LG23 Gene and Functional Analysis of Recombinant Cytochrome P450 BM3-LG23 in the Actinobacterium *Mycolicibacterium smegmatis*. Biochemistry.

[28] Miura Y., Fulco A.J. (1974). (ω–2) Hydroxylation of Fatty Acids by a Soluble System from *Bacillus megaterium*. J. Biol. Chem..

[29] Cha G.S., Ryu S.H., Ahn T., Yun C.-H. (2014). Regioselective hydroxylation of 17β-estradiol by mutants of CYP102A1 from *Bacillus megaterium*. Biotechnol. Lett..

[30] Thistlethwaite S., Jeffreys L.N., Girvan H.M., McLean K.J., Munro A.W. (2021). A promiscuous bacterial P450: The unparalleled diversity of BM3 in pharmaceutical metabolism. Int. J. Mol. Sci..

[31] Venkataraman H., de Beer S.B.A., van Bergen L.A.H., van Essen N., Geerke D.P., Vermeulen N.P.E., Commandeur J.N.M. (2012). A single active site mutation inverts stereoselectivity of 16-hydroxylation of testosterone catalyzed by engineered cytochrome P450 BM3. ChemBioChem.

[32] Dodson R.M., Kraychy S., Nicholson R.T., Mizuba S. (1962). Microbiological transformations. IX. The 1β-hydroxylation of androstenedione. J. Org. Chem..

[33] Li H., Dai W., Qin S., Li S., Yu Y., Zhang L. (2023). Regio- and stereo-selective 1β-hydroxylation of lithocholic acid by cytochrome P450 BM3 mutants. Biotechnol. Bioeng..

[34] Liu X., Wang Z.-B., Wang Y.-N., Kong J.-Q. (2016). Probing steroidal substrate specificity of cytochrome P450 BM3 variants. Molecules.

[35] Liu X., Kong J.-Q. (2017). Steroids hydroxylation catalyzed by the monooxygenase mutant 139-3 from *Bacillus megaterium* BM3. Acta Pharm. Sin. B.

[36] Lobastova T.G., Gulevskaya S.A., Sukhodolskaya G.V., Donova M.V. (2009). Dihydroxylation of dehydroepiandrosterone in positions 7α and 15α by mycelial fungi. Appl. Biochem. Microbiol..

[37] Wu Y., Li H., Zhang X.-M., Gong J.-S., Li H., Rao Z.-M., Shi Z.-H., Xu J.-S. (2015). Improvement of NADPH-dependent P450-mediated biotransformation of 7a,15a-diOH-DHEA from DHEA by a dual cosubstrate-coupled system. Steroids.

[38] Zhao Y.-Q., Liu Y.-J., Ji W.-T., Liu K., Gao B., Tao X.-Y., Zhao M., Wang F.-Q., Wei D.-Z. (2022). One-pot biosynthesis of 7β-hydroxyandrost-4-ene-3,17-dione from phytosterols by cofactor regeneration system in engineered *Mycolicibacterium neoaurum*. Microb. Cell Fact..

[39] Mohn W.W., van der Geize R., Stewart G.R., Okamoto S., Liu J. (2008). The actinobacterial *mce4* locus encodes a steroid transporter. J. Biol. Chem..

[40] Klepp L.I., Forrellad M.A., Osella A.V., Blanco F.C., Stella E.J., Bianco M.V., de la Paz Santangelo M., Sassetti C., Jackson M., Cataldi A.A. (2012). Impact of the deletion of the six mce operons in *Mycobacterium smegmatis*. Microbes Infect..

[41] Bansal-Mutalik R., Nikaido H. (2011). Quantitative lipid composition of cell envelopes of *Corynebacterium glutamicum* elucidated through reverse micelle extraction. Proc. Natl. Acad. Sci. USA.

[42] Korycka-Machala M., Ziolkowski A., Rumijowska-Galewicz A., Lisowska K., Sedlaczek L. (2001). Polycations increase the permeability of *Mycobacterium vaccae* cell envelopes to hydrophobic compounds. Microbiol..

[43] Kim S.J., Kweon O., Cerniglia C.E., Timmis K.N. (2010). Degradation of Polycyclic Aromatic Hydrocarbons by *Mycobacterium* Strain. Handbook of Hydrocarbon and Lipid Microbiology.

[44] Tekucheva D.N., Nikolayeva V.M., Karpov M.V., Timakova T.A., Shutov A.V., Donova M.V. (2022). Bioproduction of testosterone from phytosterol by *Mycolicibacterium neoaurum* strains: “one-pot”, two modes. Bioresour. Bioprocess..

[45] Egorova O., Nikolayeva V., Sukhodolskaya G., Donova M. (2009). Transformation of C_19_-steroids and testosterone production by sterol-transforming strains of *Mycobacterium* spp.. J. Mol. Catal. B Enzym..

[46] Deo A.K., Bandiera S.M. (2008). Biotransformation of lithocholic acid by rat hepatic microsomes: Metabolite analysis by liquid chromatography/mass spectrometry. Drug Metab. Dispos..

[47] Karpov M.V., Nikolaeva V.M., Fokina V.V., Shutov A.A., Kazantsev A.V., Strizhov N.I., Donova M.V. (2022). Creation and functional analysis of *Mycolicibacterium smegmatis* recombinant strains carrying the bacillary cytochromes CYP106A1 and CYP106A2 genes. Appl. Biochem. Microbiol..

[48] Daugelat S., Kowall J., Mattow J., Bumann D., Winter R., Hurwitz R., Kaufmann S.H.E. (2003). The RD1 proteins of *Mycobacterium tuberculosis*: Expression in *Mycobacterium smegmatis* and biochemical characterization. Microbes Infect..

[49] Tartoff K.D., Hobbs C.A. (1987). Improved media for growing plasmid and cosmid clones. Bethesda Res. Lab. Focus.

[50] Lobastova T.G., Gulevskaya S.A., Sukhodolskaya G.V., Turchin K.V., Donova M.V. (2007). Screening of mycelial fungi for 7α- and 7β-hydroxylase activity towards dehydroepiandrosterone. Biocatal. Biotransform..

