# Supplementary material for: Steroid Hydroxylation by Mutant Cytochrome P450 BM3-LG23 Using Two Expression Chassis
Source: Int J Mol Sci. 2025 Nov 4;26(21):10728. doi: 10.3390/ijms262110728 (PMC12608620; doi:10.3390/ijms262110728)
Supplement: Supplementary file 1 [file ijms-26-10728-s001.zip › Figure S1.pdf]

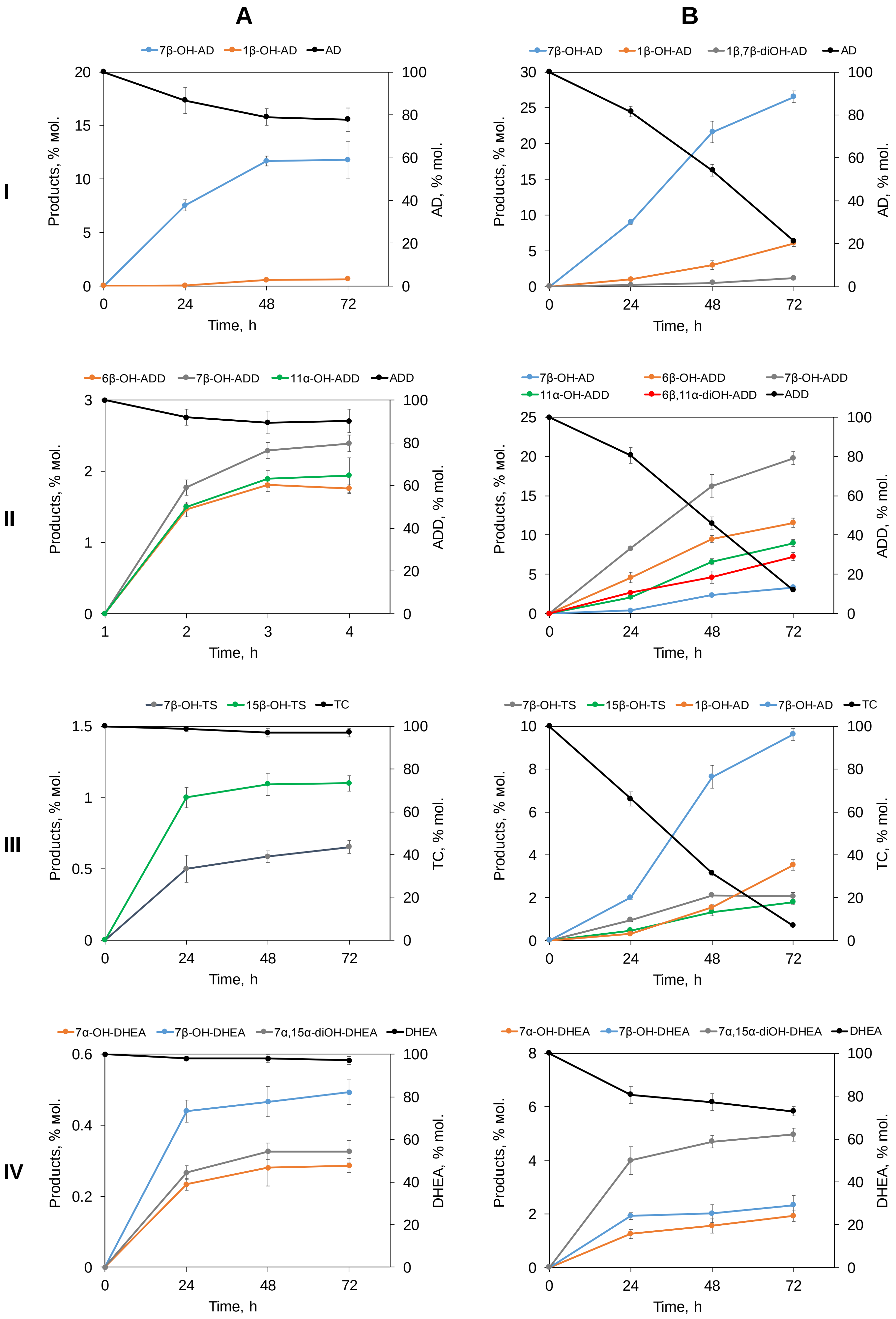

A
B
I
II
III
IV
7β-OH-AD
1β-OH-AD
AD
1β,7β-diOH-AD
6β-OH-ADD
7β-OH-ADD
11α-OH-ADD
ADD
6β,11α-diOH-ADD
7β-OH-TS
15β-OH-TS
TC
7α-OH-DHEA
7β-OH-DHEA
7α,15α-diOH-DHEA
DHEA
Products, % мol.
Products, % mol.
AD, % mol.
ADD, % mol.
TC, % mol.
DHEA, % mol.
Time, h

Figure S1. The dynamics of hydroxylation product accumulation and substrate loss during the in vivo bioconversion of steroids (**I** – AD; **II** – ADD; **III** – TS; **IV** – DHEA) by P450 BM3-LG23 in *E. coli* BL21 (DE3) (pETT1) (**A**) and *M. smegmatis* BD (pVP1) (**B**).
