# Supplementary figures and images for: Steroid Hydroxylation by Mutant Cytochrome P450 BM3-LG23 Using Two Expression Chassis
Source: Int J Mol Sci. 2025 Nov 4;26(21):10728. doi: 10.3390/ijms262110728 (PMC12608620; doi:10.3390/ijms262110728)

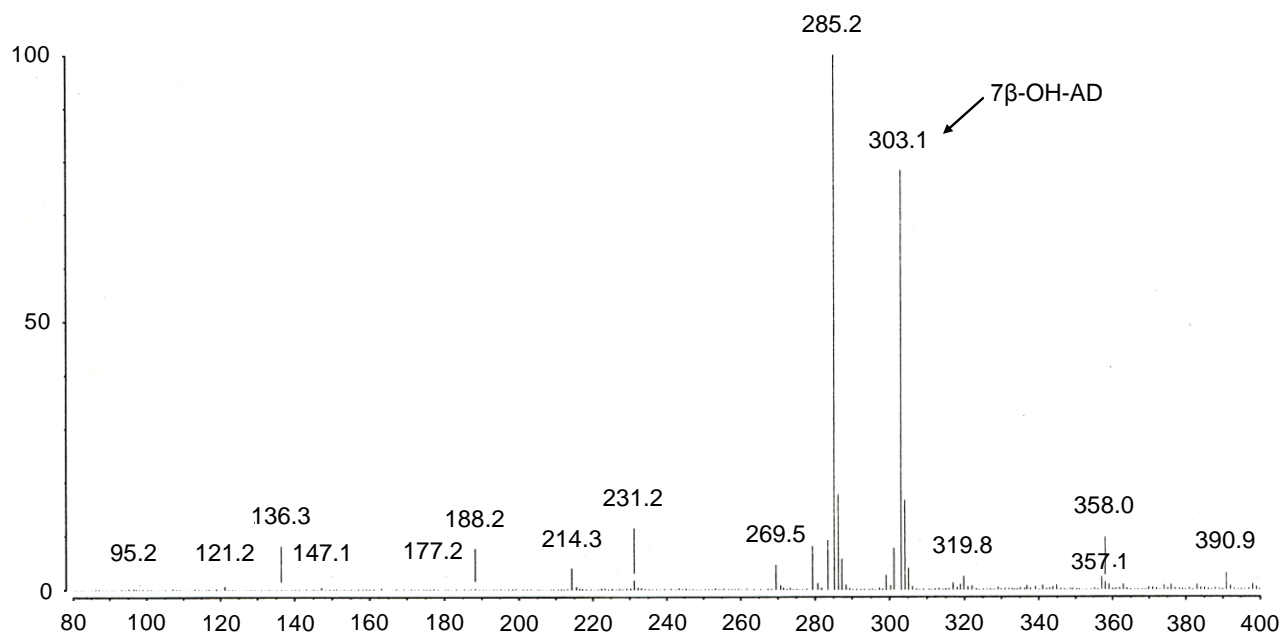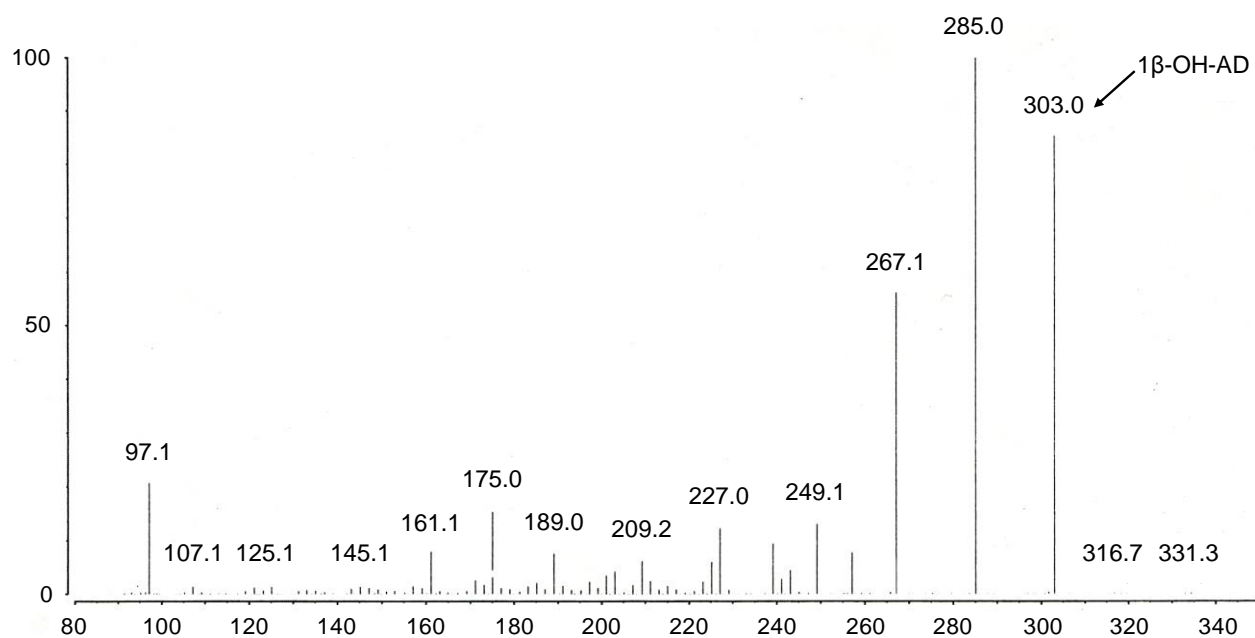

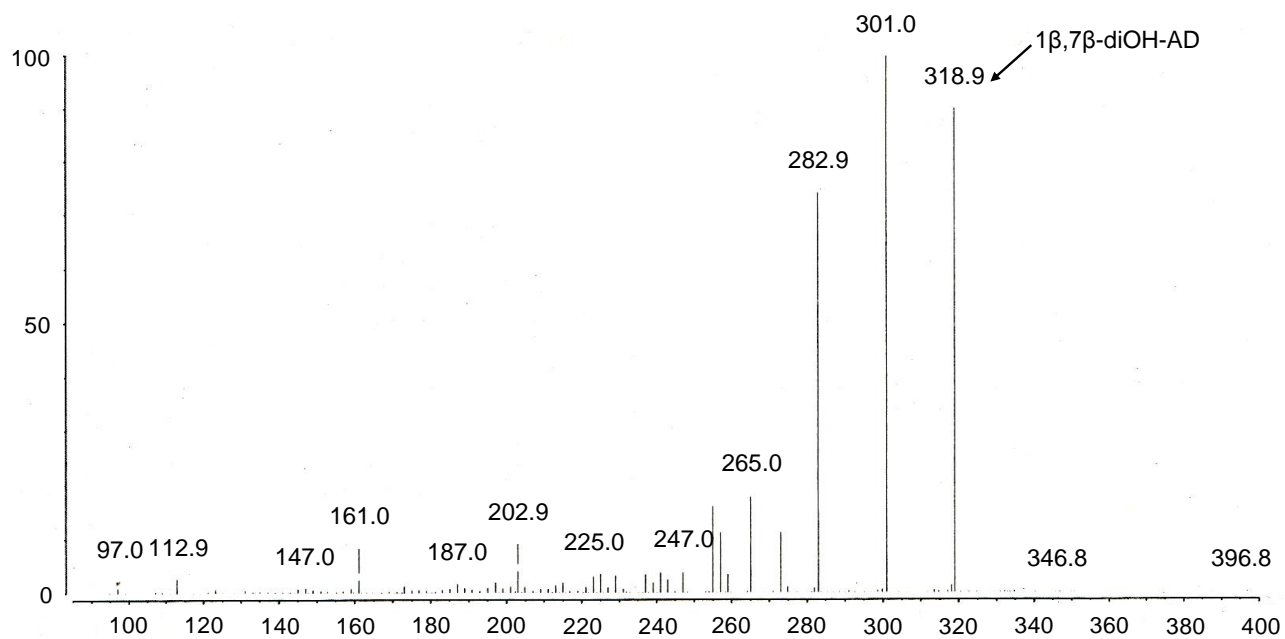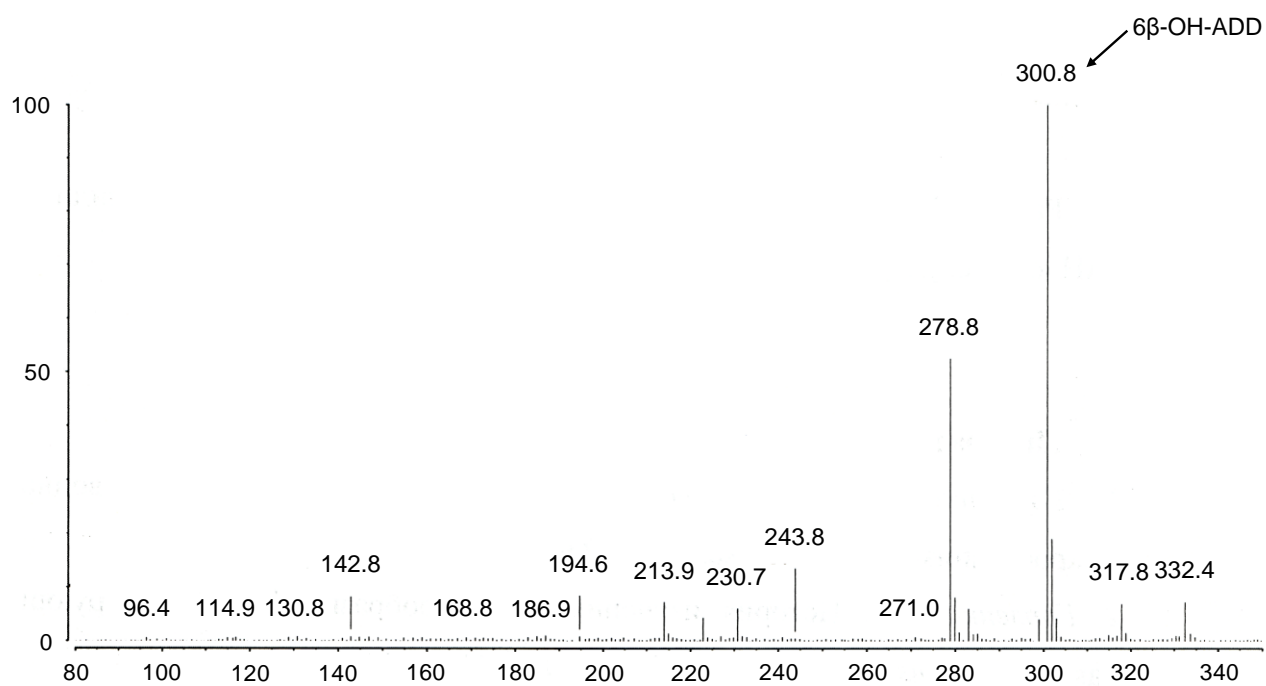

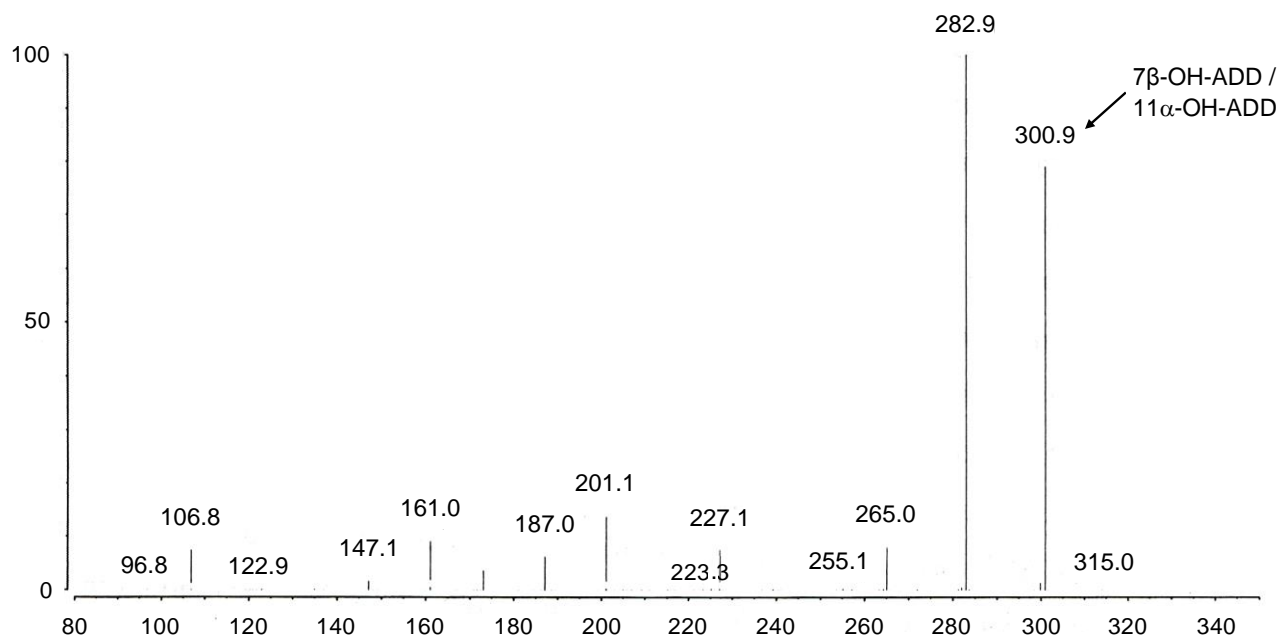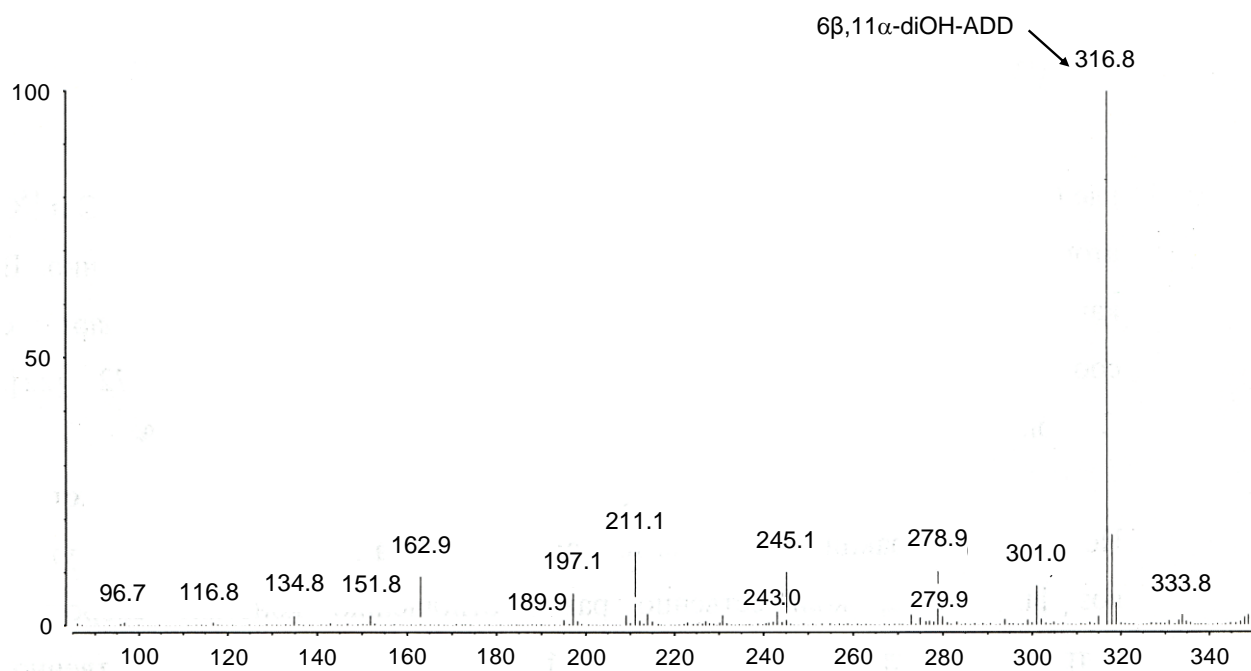

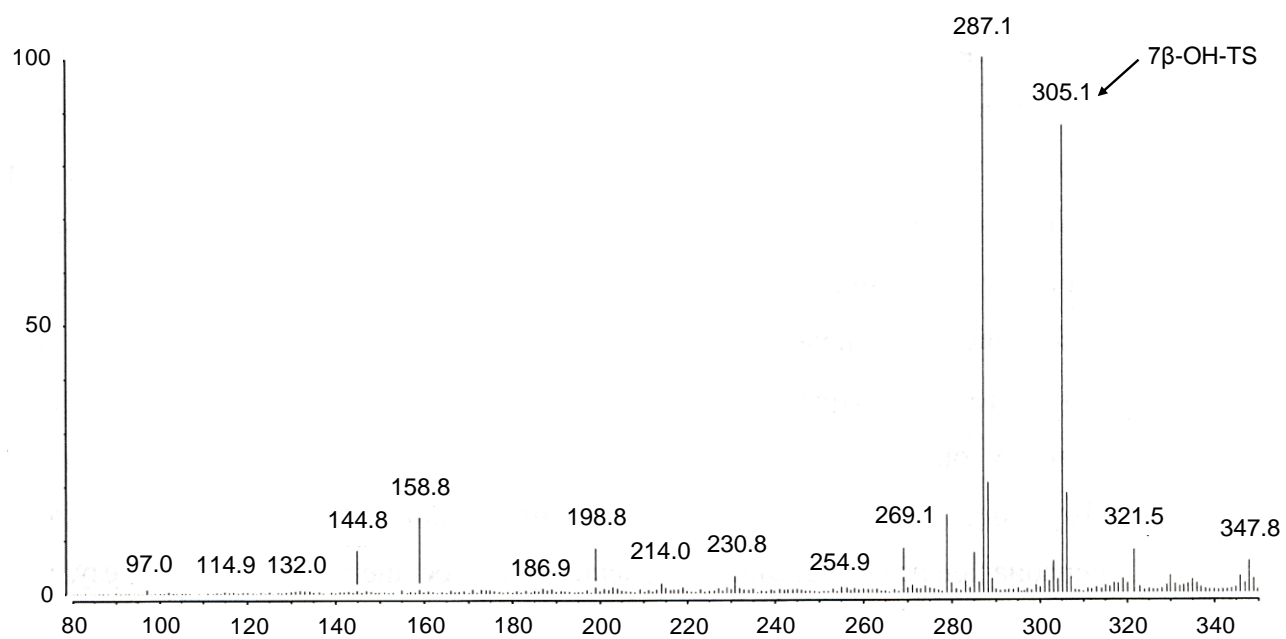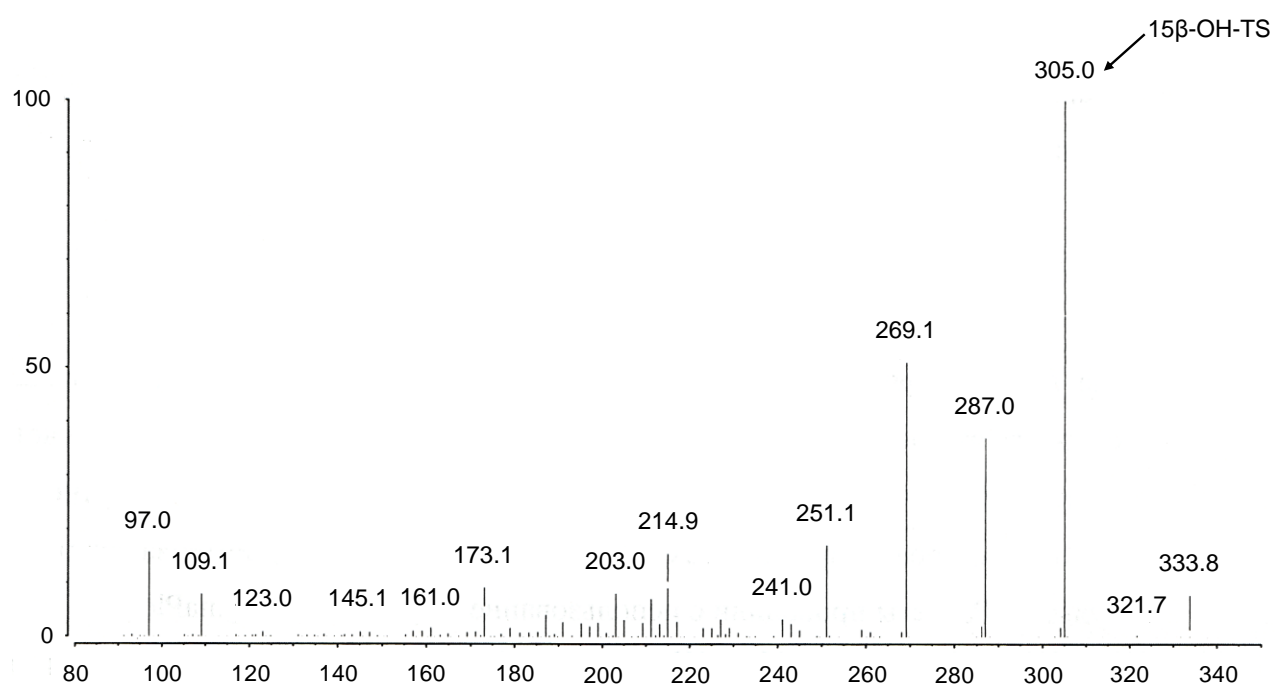

**Figure S2.** The MS/MS spectra of the in vivo steroid bioconversion by P450 BM3-LG23.

Supplement: Supplementary file 1 [file ijms-26-10728-s001.zip › Figure S2.pdf]
